# The Impact of Digital Technology–Based Exercise Combined With Dietary Intervention on Body Composition in College Students With Obesity: Prospective Study

**DOI:** 10.2196/65868

**Published:** 2025-06-02

**Authors:** Chengyuan Hu, Zixin Lv, Jieping Zhu, Chunyuan Lai, Dongjuan Guo, Maolin Chen, Xiaoyan Cheng, Mingxin Rao, Xinyou Zhou, Liqiang Su

**Affiliations:** 1 School of Physical Education Jiangxi Normal University Nanchang China

**Keywords:** digital health, diet, obesity, college students, exercise, body composition

## Abstract

**Background:**

Lifestyle interventions are a critical component of weight loss programs, yet digital, personalized, and theory- and evidence-based lifestyle interventions remain limited.

**Objective:**

This study aimed to investigate the effects of a combination of various dietary approaches and digital technology–based exercise on the body composition of college students with obesity.

**Methods:**

A total of 129 college students with obesity (mean age 18.3, SD 0.7 years; mean weight 89.9, SD 13.6 kg; mean BMI 30.6, SD 3.3 kg/m^2^) were initially recruited for this study. After excluding 2 participants, 127 students with obesity were ultimately included in the statistical analysis. An 8-week weight loss intervention was conducted with the students, combining exercise and various digitally supported dietary approaches. Body composition indicators (muscle mass and fat mass) were assessed before and after the intervention. Participants were divided into 3 experimental groups (twice-weekly fasting [TWF], low-calorie diet [LCD], and time-restricted feeding [TRF]). Between-group comparisons were made using a 1-way ANOVA, while within-group comparisons used a repeated-measures ANOVA. Linear mixed-effects models were used to examine the interaction effects between sex and time, as well as between sex and group.

**Results:**

All groups showed significant decreases in weight and BMI, and the TRF group also showed a significant decrease in BMI (*P*=.002), but there were significant sex differences. The male TWF group showed the largest decrease in weight (mean difference [MD] −4.86 kg; *P*<.001), BMI (MD −1.1 kg/m^2^; *P*<.001), visceral fat mass (MD −0.607 kg; *P=*.003) and subcutaneous fat mass (MD −1.987 kg; *P*<.001) at 8 weeks. Improvements in weight (MD −5.662 kg; *P*<.001) and BMI (MD −1.587 kg/m^2^; *P*<.001) were more pronounced in the LCD group of female participants (*P*<.001). Muscle mass declined significantly in male participants in the TRF group at 4 weeks (*P*<.001) but stabilized at 8 weeks (*P*=.87). Linear mixed effects models showed that the sex and diet interaction significantly affected subcutaneous fat mass (*P*=.02). The effect of TRF on muscle mass in male participants peaked at 4 weeks (*P*<.001), with no significant difference from the control group at 8 weeks (*P*=.91).

**Conclusions:**

This study demonstrated that 3 diet-combined exercise regimens produced sex-specific improvements in body composition in college students with obesity. Male participants achieved maximum visceral fat mass loss after 8 weeks with TWF combined with exercise, whereas female participants achieved greater total body fat loss with LCD combined with exercise. The effectiveness of the closed-loop monitoring-feedback behavior modification was verified by digital technology through intelligent monitoring to improve dietary compliance and a real-time feedback mechanism to enhance the effect of the intervention. The sex and diet interaction significantly affected subcutaneous fat mass; women who used LCD and TRF needed additional protein supplementation. Digital technology shows great potential in obesity management and is worth promoting.

## Introduction

### Background

Over the past 4 decades, there has been a dramatic increase in obesity and related diseases, with approximately 50% of adults in China having overweight or obesity [1]. By 2030, the number of individuals with overweight or obesity in China is predicted to increase to 790 million, and the medical costs associated with overweight and obesity are expected to reach US $61 billion [2]. Overweight or obesity is also a major risk factor for many noncommunicable diseases, including cardiovascular diseases [3], musculoskeletal disorders [4], diabetes [5], and cancers (breast, uterine, prostate, liver, and kidney) [6-8]. The World Health Organization states that overweight and obesity are the fifth leading cause of death worldwide and have become a serious social issue, emphasizing the urgency of scientific weight loss interventions. Currently, mainstream scientific weight loss strategies focus on health guidance, including face-to-face counseling and paper-based recording methods [9-12]. Although face-to-face guidance can lead to substantial weight loss, the high resource and cost investment cannot be overlooked. Therefore, exploring an effective, low-cost, and low-energy-consuming weight loss strategy is particularly urgent. The rise of digital technology offers new insights into addressing this issue, giving rise to numerous digital-based weight loss methods that can greatly optimize health care services [13-15].

In the health sector, digital technology has emerged as an innovative method for weight loss, harnessing advanced information and communication technologies that effectively meet the demands of weight reduction [16-18]. Systematic reviews indicate [19] that compared to general interventions, digital-based interventions are more effective in supporting weight loss. This technology can provide personalized services to participants through a variety of methods, each with varying degrees of effectiveness. For instance, smartphones [20-23], with their intuitive and appealing nature, not only facilitate participants’ self-monitoring of diet and physical activity (PA) but also play a key role in promoting behavior change related to weight management, making them critical in weight loss practices.

Another advantage of digital technology interventions is their ability to provide timely feedback. Currently, self-monitoring of diet, PA, and weight is considered one of the effective strategies in most weight loss interventions [24,25]. Although self-monitoring has been proven effective in both theory and practice, it often fails to provide immediate feedback on behavioral information such as diet and PA, which can lead to a decrease in compliance. Research by Burke et al [26] showed that compliance with self-monitoring substantially decreases over time during participation in weight loss behavioral treatment programs. In addition, the combination of self-monitoring and feedback can enhance behavior change. The synchronization of data via smartphones eliminates the need for manual recording of exercise and weight, thereby reducing the burden on participants and increasing compliance.

While the combination of self-monitoring and feedback has shown substantial effectiveness in weight loss practices, the lack of theoretical support may undermine its long-term outcomes and user compliance. For instance, digitally enabled platforms like Voy incorporate behavioral change theories, such as the social cognitive theory [27], and facilitate self-monitoring, goal setting, and feedback mechanisms, encouraging patients to actively manage their health and well-being in the community and other settings. These platforms not only provide technological support but also ensure the scientific validity and effectiveness of interventions through theoretical guidance. Behavioral activation complements weight loss interventions by helping individuals identify and engage in positive, goal-oriented activities that align with their health objectives. It emphasizes breaking the cycle of avoidance or inactivity often associated with obesity, replacing these behaviors with structured, rewarding actions like regular PA, healthy eating, and consistent self-monitoring [28]. By focusing on small, achievable steps, behavioral activation builds motivation and self-efficacy, which are crucial for sustained adherence to treatment protocols. When combined with digital tools, such as goal-setting features and real-time feedback, this approach empowers individuals to take ownership of their weight management journey, enhancing both engagement and long-term outcomes [29].

Current research indicates that there is no substantial difference in weight loss outcomes between self-monitoring combined with feedback and self-monitoring alone. However, it is noteworthy that participants who used the feedback mechanism demonstrated better compliance and more effective weight loss [30]. These studies have certain limitations: they did not provide specific dietary plans or exercise programs, nor did they conduct phase-based or sex-specific comparisons. These findings offer important insights into future research, suggesting that structured intervention programs should be explored for their effects across different populations. In early research, the critical variable of sex was often overlooked, primarily due to the substantial underrepresentation of female participants in both preclinical and clinical studies [31,32]. The National Institutes of Health implemented a policy requiring all National Institutes of Health–funded research projects to include both male and female participants [32]. This policy has driven in-depth exploration of sex differences across various research fields, and obesity research is no exceptions. Currently, significant evidence demonstrates that the pathogenesis, risk factors, and clinical implications of obesity all exhibit substantial sex-specific differences [33,34]. Therefore, systematically investigating sex-specific outcomes in research is fully justified by scientific evidence and necessity.

### Objectives and Hypotheses

This study used digital technology to conduct an 8-week intervention on college students with obesity through a combination of exercise and different dietary approaches, on the basis of behavior change theories such as social cognitive theory, to improve the health status of college students with obesity.

The study observed improvements in muscle indices (such as lean body mass [LBM] and muscle volume, among others) and fat indices (such as visceral fat mass and subcutaneous fat mass, among others). In addition, this research comparatively analyzed the effects of combined exercise and different dietary approaches on these indices and their subindicators, with the goal of providing personalized weight loss solutions for individuals with obesity, further enriching the theoretical understanding of dietary approaches for obesity, and offering scientific evidence for the clinical treatment of obesity. Specifically, we aimed to achieve the following 2 objectives:

1. To evaluate the impact of a combination of various dietary approaches and exercise, based on digital technology, on the body composition of college students with obesity.
2. To assess whether the addition of self-monitoring and feedback to the combination of various dietary approaches and exercise could enhance participation and compliance.

We hypothesized that participants following these 3 dietary approaches would experience at least a small to moderate improvement in body composition indicators, including muscle and fat mass. In addition, we hypothesized that interventions incorporating digital technology for self-monitoring and feedback, which are implemented based on behavioral change theories such as social cognitive theory [27], will lead to higher compliance and more substantial weight loss outcomes. By integrating these theories, the interventions effectively promoted self-monitoring, goal setting, and feedback mechanisms, thereby enhancing the overall effectiveness of the dietary approaches.

## Methods

### Study Design

The study was conducted in Nanchang, China, from October 2023 to December 2023. Our team provided an 8-week intervention. Three groups used a WeChat (Tencent Holdings Ltd) Mini program for self-monitoring and feedback.

### Ethical Considerations

The study was approved by the Ethics Committee of Jiangxi Normal University with the ethical code IRB-JXNU-20231027 and was registered at the Chinese Clinical Trial Registry (ChiCTR2400093865). All data, especially the users’ information on the Mini program, were identified by code numbers to ensure the confidentiality of the participants’ information. No compensation was provided to the participants.

### Sample Size

To determine the sample size for this study, calculations were conducted based on effect size, significance level, and statistical power. Referring to the mean differences (MDs) and SDs in subsequent sections, an effect size of 0.45 was assumed, with a significance level (α) of .05 and statistical power (1–β) of 0.80. Based on the standard normal distribution table, Zα/2 (2-tailed test) was 1.96, and Zβ was 0.84. The formula calculation yielded a total sample size of 81 participants, with 27 participants per group. Therefore, the study design of 27 participants per group, totaling 81 participants, met the requirements of an effect size of 0.45, a significance level of .05, and a statistical power of 0.80, ensuring the reliability and statistical validity of the research results.

### Participants

This study recruited students with a BMI ≥28 kg/m^2^ through a weight loss class for college students at Jiangxi Normal University in October 2023. A total of 144 students were enrolled, with 129 meeting the inclusion criteria. Two participants withdrew during the study, resulting in a final sample size of 127 individuals (n=62, 48.8% male and n=65, 51.2% female). Of 127 participants, the low-calorie diet (LCD) group comprised 51 (40.2%) participants, the twice-weekly fasting (TWF) group comprised 45 (35.4%) participants, and the time-restricted feeding (TRF) group comprised 31 (24.4%) participants. The inclusion and exclusion criteria are given in Textbox 1.

**Inclusion and exclusion criteria.**

**Inclusion criteria**

- BMI ≥28 kg/m^2^
- Absence of disease
- Voluntary participation in the intervention study with good compliance and signing of the informed consent form

**Exclusion criteria**

- BMI <28 kg/m^2^
- Individuals with restricted physical activity (eg, disability)
- Those with diagnosed chronic diseases and currently on medication
- Participants who used other dietary plans in the past 3 months
- Those with low compliance who withdraw from the intervention in the middle of the study

### Intervention

**Figure 1:**
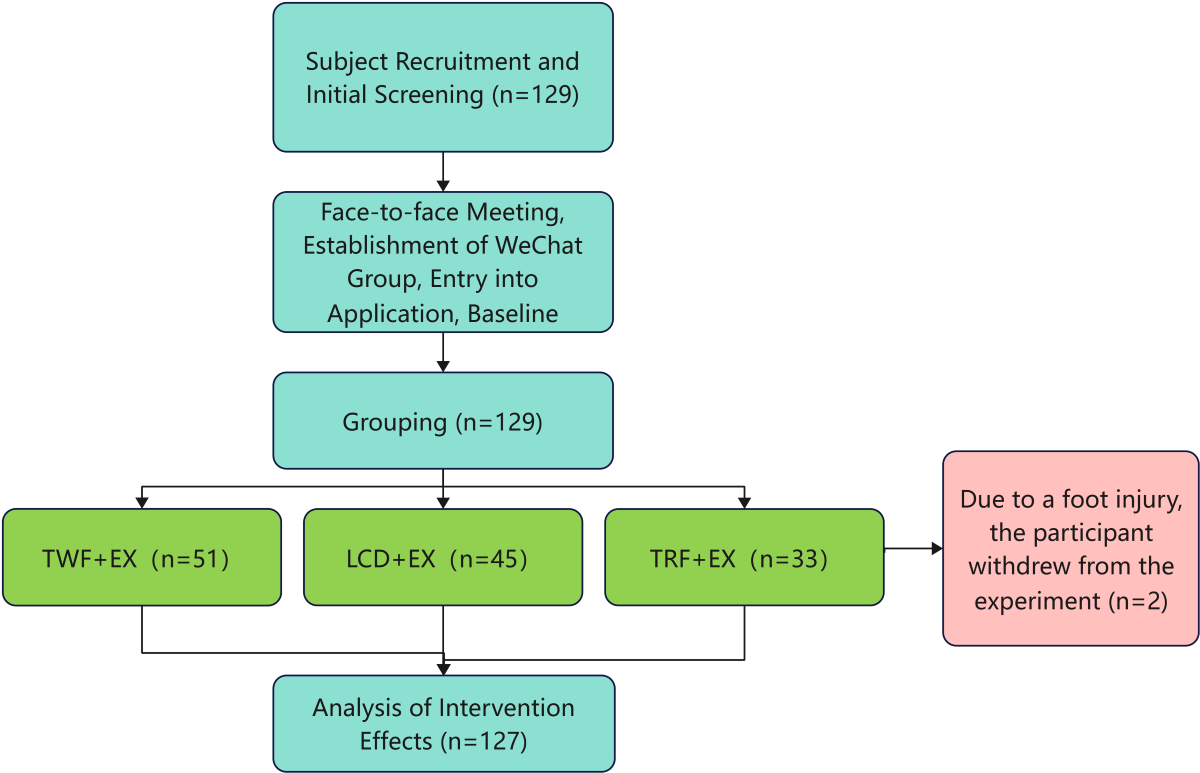
Flowchart of the study. EX: exercise; LCD: low-calorie diet; TRF: time-restricted feeding; TWF: twice-weekly fasting.

The study used a digital technology intervention approach as a medium, using the WeChat app for self-monitoring and feedback, to investigate the impact of a combination of 3 dietary methods and exercise on the body composition of college students with obesity. The intervention involved an 8-week experimental study. In total, 129 participants were recruited and initially screened. Following a face-to-face meeting, the establishment of a WeChat group, and baseline assessments, participants voluntarily chose to join 1 of 3 intervention groups: TWF+exercise (51/129, 39.5%), LCD+exercise (45/129, 34.9%), or TRF+exercise (33/129, 25.6%). During the experiment, 2 (1.6%) participants of 129 withdrew due to foot injuries. Ultimately, 127 participants completed the intervention effectiveness analysis to evaluate the impact of different dietary and exercise regimens on health indicators (Figure 1). The intervention incorporated knowledge, exercise, diet, self-monitoring, and feedback, as detailed in Multimedia Appendix 1 [35-37] and the specific intervention protocol is outlined in Multimedia Appendix 2.

### Body Composition Measurement

Participants were assessed for body composition at baseline, 4, and 8 weeks using the Tanita MC-980U Plus (Tanita Corp) multifrequency segmental body composition analyzer [38]. They removed their socks and stood on the foot plates, holding the electrode handles, maintaining a fixed posture during the test. Participants entered their height and clothing weight (0.5 kg) as prompted and initiated the test according to the instrument’s instructions. Test data were automatically recorded into the computer for storage. Measurement indices included muscle parameters (LBM and muscle mass) and fat parameters (body weight, BMI, visceral fat mass, and subcutaneous fat mass).

### Compliance

To deeply analyze the differences in compliance between different dietary patterns, we divided the study participants into different groups based on their individual dietary choices. Within each dietary group, we quantified compliance by 2 methods: taking photos during the dietary fasting period and counting the number of exercise check-ins.

In terms of dietary assessment, the study was designed based on relevant literature [39-41]. Participants were required to upload photos of their 3 daily meals once a day, along with textual descriptions of the food and their daily subjective feelings of satiety, to minimize bias. To quantify compliance, each successful submission, after review of the photo, text, and subjective feedback, was awarded 0.2 points. When the number of uploads reached the upper limit per week, they could get a full score of 1 point. Over the entire 8-week study period, the full dietary compliance score was 8 points. A higher score indicated better compliance during the dietary fasting period.

For exercise, we set the requirement of 2 exercise check-ins per week. Each check-in was worth 0.5 points, and the full score for weekly exercise check-ins was also 1 point. Over the 8-week study, the cumulative full score for exercise check-ins was 8 points. Similarly, a higher score meant better compliance in terms of exercise (the detailed statistics on compliance are presented in Multimedia Appendix 3).

### Bias

Self-selected groups may introduce bias. To mitigate this, we implemented the following measures: first, all eligible participants who initiated the program within the study period were included to ensure the sample’s representativeness. Second, we enhanced data accuracy through regular reminders and data validation checks, performing repeated validation checks to identify and address implausible values. In addition, we standardized training for assessors to ensure consistency in the evaluation process, thereby minimizing bias. We also reduced bias by examining baseline group differences and conducting sex-stratified analyses. These measures collectively improved data validity and the reliability of the study findings.

### Statistical Analysis

Data were analyzed using SPSS (version 26.0; IBM Corp) statistical software. Outliers were identified using box plots. The distribution of continuous variables was assessed using graphical methods (eg, histograms and *Q*-*Q* plots) and supplemented with the Shapiro-Wilk test. Continuous variables with a normal distribution are presented as mean and SD and were analyzed using 1-way ANOVAs across the 3 groups. For variables that did not meet the assumption of normality, the median and IQR were reported, and the Kruskal-Wallis test was used for comparisons between the 3 groups. Comparisons between groups were performed using 1-way ANOVA. Within-group comparisons were conducted using repeated measures ANOVA. Comparisons between male and female participants were performed using an independent samples 1-tailed *t* test. The significance level was set at α=.05, and a *P* value <.05 was considered statistically significant.

To account for the longitudinal structure of the data, linear mixed-effects models were used in our study. In this model, diet groups (3 types), time points (0, 4, and 8 weeks), and sex were included as fixed effects, including interactions such as diet and time and sex and diet, with random effects accounting for individual differences, which could help us explore group differences and within-person changes over time. Advantages over original methods were that it reduces type 1 error risk by modeling dependencies between repeated measures and provides more precise estimates of intervention effects compared to separate ANOVAs. Compared to the original approach, linear mixed models reduce type 1 error risk by modeling dependencies between repeated measures and provide more precise estimates of intervention effects than separate ANOVA.

## Results

### Participant Characteristics

A total of 127 individuals were enrolled, including 62 male individuals (n=18, 14.2% in the TWF group; n=29, 22.8% in the LCD group; and n=15, 11.8% in the TRF group) and 65 female individuals (n=33, 26% in the TWF group; n=16, 12.6% in the LCD group; and n=16, 12.6% in the TRF group). Due to sex differences, a sex-specific analysis was conducted. Comparison of baseline characteristics among the 3 groups revealed no statistically significant differences, indicating that the 3 groups were comparable. Normality and homogeneity of variance tests were performed at different time points for the 3 groups, and the results showed that all 3 groups followed a normal distribution with homogeneity of variance (all *P*>.05). In the TWF group, the average energy intake was 525 (SD 18.2) kcal for male participants and 465 kcal for female participants. In the LCD group, the average energy intake was 784.1 kcal for male participants and 727.6 (SD 49.8) kcal for female participants. Due to the 8-hour TRF and 16-hour fasting regimen in the TRF group, it was difficult to accurately quantify their energy intake (Tables 1 and 2).

**Table 1:** Basic participant characteristics of the 3 groups for male participants.

Index	TWF^a^+exercise (n=18), mean (SD)	LCD^b^+exercise (n=29), mean (SD)	TRF^c^+exercise (n=15), mean (SD)	*F* test (*df*) or *t* test (*df*)	*P* value
Age (y)	18.22 (0.73)	18.45 (0.69)	18.3 (0.82)	0.538 (2, 59)^d^	.59
Body weight (kg)	95.75 (11.4)	97.46 (12.87)	98.17 (16.79)	0.146 (2, 59)^d^	.86
BMI (kg/m^2^)	30.37 (2.98)	31.09 (3.89)	31.25 (3.64)	0.308 (2, 59)^d^	.74
Lean body mass (kg)	63.4 (5.07)	65 (4.43)	63.6 (6.79)	0.638 (2, 59)^d^	.53
Muscle mass (kg)	60.12 (4.82)	61.64 (4.21)	60.32 (6.45)	0.64 (2, 59)^d^	.53
Visceral fat mass (kg)	6.01 (1.94)	6.06 (2.78)	6.92 (3.32)	0.602 (2, 59)^d^	.55
Subcutaneous fat mass (kg)	25.15 (5.11)	25.06 (7.1)	26.39 (7.33)	0.218 (2, 59)^d^	.80
Average energy intake (kcal)	525 (18.2)	784.1 (49.8)	—^e^	−21.4 (45)^f^	<.001

^a^TWF: twice-weekly fasting.

^b^LCD: low-calorie diet.

^c^TRF: time-restricted feeding.

^d^*F* test.

^e^Not available.

^f^*t* test.

**Table 2:** Basic participant characteristics of the 3 groups for female participants.

Index	TWF^a^+exercise (n=33), mean (SD)	LCD^b^+exercise (n=16), mean (SD)	TRF^c^+exercise (n=16), mean (SD)	*F* test (*df*) or *t* test (*df*)	*P* value
Age (y)	18.64 (0.82)	18.06 (0.44)	18.25 (0.68)	3.953 (2, 62)^d^	.02
Body weight (kg)	82.91 (10.08)	83.55 (10.3)	83.13 (10.37)	0.021 (2, 62)^d^	.98
BMI(kg/m^2^)	30.83 (3.2)	29.53 (2.99)	30.16 (3.01)	0.985 (2, 62)^d^	.38
Lean body mass (kg)	45.08 (3.05)	46.33 (3.74)	45.21 (3.84)	0.755 (2, 62)^d^	.47
Muscle mass (kg)	42.29 (2.78)	43.44 (3.42)	42.41 (3.51)	0.771 (2, 62)^d^	.47
Visceral fat mass (kg)	5.92 (2.33)	6.11 (2.25)	6 (2.31)	0.04 (2, 62)^d^	.96
Subcutaneous fat mass (kg)	31.12 (5.07)	30.79 (6)	30.46 (5.72)	0.081 (2, 62)^d^	.92
Average energy intake (kcal)	465.3 (57.6)	727.6 (45.2)	—^e^	−16.0 (47)^f^	<.001

^a^TWF: twice-weekly fasting.

^b^LCD: low-calorie diet.

^c^TRF: time-restricted feeding.

^d^*F* test.

^e^Not available.

^f^*t* test.

### Comparison of Muscle Indices Before and After the Intervention

For muscle parameters, the results of 1-way ANOVA showed no significant differences (at *P*>.05) among the groups. Compared to baseline, all 3 groups showed a decrease in LBM and muscle mass at 8 weeks. In particular, male participants in the TRF group showed a significant reduction in LBM (*P*<.001) and muscle mass (*P*<.001) at 4 weeks. However, no further significant decline was observed at 8 weeks compared to 4 weeks (LBM: *P*=.91; muscle mass: *P*=.87). Detailed data are shown in Figure S1 in Multimedia Appendix 4 and Table 3.

**Table 3:** Comparison of muscle indices among the 3 groups after the intervention.

Sex, index, and group				0 wk, mean (SD)	4 wk, mean (SD)	8 wk, mean (SD)	*P* value (0 wk and 4 wk)	*P* value (0 wk and 8 wk)	*P* value (8 wk and 4 wk)	MD^a^ (8 wk to 0 wk)	*F* test (*df*)	*P* value
**Male (n=62)**												
		**Lean body mass (kg)**										
			TWF^b^+exercise	63.4 (5.1)	63 (5)	62.3 (5.2)^c,d^	.11	.003	.01	−1.107	4.713 (2, 58)	.01
			LCD^e^+exercise	65 (4.43)	64.5 (4.5)^c^	63.8 (4.9)^c,d^	.009	<.001	.001	−0.773	10.049 (2, 58)	<.001
			TRF^f^+exercise	63.6 (6.8)	62.3 (6.6)^c^	62.3 (6.8)^c^	<.001	.001	.91	−1.293	12.275 (2, 58)	<.001
			*F* test (*df*)	0.638 (2, 59)	0.971 (2, 59)	0.532 (2, 59)	—^g^	—	—	—	—	—
			*P* value	.53	.38	.59	—	—	—	—	—	—
		**Muscle mass (kg)**										
			TWF+exercise	60.1 (4.8)	59.8 (4.8)	59.1 (5.0)^c^	.10	.004	.02	−1.040	4.575 (2, 58)	.01
			LCD+exercise	61.7 (4.2)	61.2 (4.3)^c^	60.5 (4.7)^c,d^	.009	<.001	.001	−0.727	10.021 (2, 58)	<.001
			TRF+exercise	60.3 (6.5)	59.1 (6.3)^c^	59.1 (6.5)^c^	<.001	.001	.87	−1.247	12.389 (2, 58)	<.001
			*F* test (*df*)	0.640 (2, 59)	0.973 (2, 59)	0.538 (2, 59)	—	—	—	—	—	—
			*P* value	.53	.38	.59	—	—	—	—	—	—
**Female (n=65)**												
		**Lean body mass (kg)**										
			TWF+exercise	45.1 (3.1)^h^	44.4 (3.1)^i^	45.2 (4.9)^j^	<.001	.76	.07	−0.381	11.309 (2, 61)	.001
			LCD+exercise	46.3 (3.7)^h^	45.9 (3.6)^i^	45.8 (3.8)^j^	.03	.39	.88	−0.550	1.622 (2, 61)	.23
			TRF+exercise	45.2 (3.8)^h^	44.9 (3.8)^i^	44.9 (4.12)^j^	.12	.66	.94	−0.275	1.689 (2, 61)	.22
			*F* test (*df*)	0.755 (2, 62)	0.968 (2, 62)	0.151 (2, 62)	—	—	—	—	—	—
			*P* value	.47	.39	.86	—	—	—	—	—	—
		**Muscle mass (kg)**										
			TWF+exercise	42.3 (2.8)^h^	41.7 (2.8)^i^	42.5 (4.6)^j^	<.001	.7	.08	−0.350	11.211 (2, 61)	<.001
			LCD+exercise	43.4 (3.4)^h^	43 (3.3)^i^	43 (3.5)^j^	.03	.42	.89	−0.494	2.480 (2, 61)	.09
			TRF+exercise	42.4 (3.5)^h^	42.1 (3.5)^i^	42.2 (3.8)^j^	.13	.68	.96	−0.256	1.176 (2, 61)	.32
			*F* test (*df*)	0.771 (2, 62)	0.991 (2, 62)	0.149 (2, 62)	—	—	—	—	—	—
			*P* value	.47	.38	.86	—	—	—	—	—	—

^a^MD: mean difference.

^b^TWF: twice-weekly fasting.

^c^Within-group comparisons: indicates *P*<.05 compared to baseline.

^d^Within-group comparisons: indicates *P<*.05 compared to 4 weeks.

^e^LCD: low-calorie diet.

^f^TRF: time-restricted feeding.

^g^Not applicable.

^h^Sex comparisons: indicates *P<*.05 compared to male participant baseline.

^i^Sex comparisons: indicates *P<*.05 compared to 4 weeks in male participants.

^j^Sex comparisons: indicates *P<*.05 compared to 8 weeks in male participants.

### Comparison of Fat Indices Before and After the Intervention

All groups showed significant decreases (*P*<.001) in weight and BMI, and the TRF group also showed a significant decrease in BMI (*P*=.002). Compared with baseline, the body weight (*P*<.001; MD −4.86), BMI (*P*<.001; MD −1.1), visceral fat mass (*P*=.003; MD −0.607), and subcutaneous fat mass (*P*<.001; MD −1.987) of male participants in the TWF group significantly decreased at 8 weeks, while the body weight (*P*<.001; MD −5.662), BMI (*P*<.001; MD −1.587), visceral fat mass (*P*=.001; MD −1.031), and subcutaneous fat mass (*P*=.001; MD −3.638) of female participants in the LCD group significantly decreased at 8 weeks. Detailed data are shown in Figure S2 in Multimedia Appendix 4 and Table 4.

**Table 4:** Comparison of fat indices among the 3 groups after the intervention.

Sex, index, and group				0 wk, mean (SD)		4 wk, mean (SD)		8 wk, mean (SD)		*P* value (0 wk and 4 wk)		*P* value (0 wk and 8 wk)		*P* value (8 wk and 4 wk)		MD^a^ (8 wk and 0 wk)		*F* test (*df*)		*P* value	
**Male (n=62)**																					
		**Body weight (kg)**																			
			TWF^b^+exercise		95.8 (11.4)		92.3 (11.1)^c^		90.5 (11.2)^c,d^		<.001		<.001		0.001		−4.86		20.418 (2, 58)		<.001
			LCD^e^+exercise		97.5 (12.9)		94.8 (13.6)^c^		93.1 (14.1)^c,d^		<001		<.001		<.001		−3.39		27.853 (2, 58)		<.001
			TRF^f^+exercise		98.2 (16.8)		95.3 (17.4)^c^		93.3 (17)^c,d^		<.001		<.001		<.001		−4.83		17.373 (2, 58)		<.001
			*F* test (*df*)		0.146 (2, 59)		0.235 (2, 59)		0.15 (2, 59)		—^g^		—		—		—		—		—
			*P* value		.86		.79		.86		—		—		—		—		—		—
		**BMI (kg/m** ^2^ **)**																			
			TWF+exercise		30.4 (3)		29.7 (3.1)^c^		29 (3.1)^c,d^		.002		<.001		.003		−1.1		9.516(2,58)		<.001
			LCD+exercise		31.1 (3.9)		30.7 (4.1)^c^		30.1 (4.3)^c,d^		.01		<.001		<.001		−0.73		18.905(2,58)		<.001
			TRF+exercise		31.3 (3.6)		30.9 (4)		30.1 (3.9)^c,d^		.12		<.001		<.001		−1.1		13.590 (2, 58)		<.001
			*F* test (*df*)		0.308 (2, 59)		0.484 (2, 59)		0.257 (2, 59)		—		—		—		—		—		—
			*P* value		.74		.62		.77		—		—		—		—		—		—
		**Visceral fat mass**																			
			TWF+exercise		6.01 (1.9)		5.59 (1.9)^c^		5.46 (1.9)^c^		.009		.003		.19		−0.607		4.828 (2, 58)		.01
			LCD+exercise		6.06 (2.8)		6.02 (3.1)		5.77 (3.1)		.78		.04		.003		−0.04		4.876 (2, 58)		.01
			TRF+exercise		6.92 (3.3)		6.93 (3.8)		6.46 (3.6)		.97		.02		.07		−0.460		8.223 (2, 58)		.001
			*F* test (*df*)		0.602 (2, 59)		0.832 (2, 59)		0.486 (2, 59)		—		—		—		—		—		—
			*P* value		.55		.44		.62		—		—		—		—		—		—
		**Subcutaneous fat** **mass**																			
			TWF+exercise		25.2 (5.1)		23.8 (5.3)^c^		23.2 (5.2)^c,d^		<.001		<.001		.03		−1.987		15.035 (2, 58)		<.001
			LCD+exercise		25.1 (7.1)		24.2 (7.2)^c^		23.5 (7.2)^c,d^		<.001		<.001		.002		−1.387		14.736 (2, 58)		<.001
			TRF+exercise		26.4 (7.3)		26.0 (7.9)		24.6 (7.4)^c,d^		.21		<.001		<.001		−1.820		14.797		<.001
			*F* test (*df*)		0.218 (2, 59)		0.466 (2, 59)		0.179 (2, 59)		—		—		—		—		—		—
			*P* value		.8		.63		.84		—		—		—		—		—		—
**Female (n=65)**																					
		**Body weight (kg)**																			
			TWF+exercise		82.9 (10.1)^h^		80.5 (10.21)^c,i^		78.8 (10.4)^c,d,j^		<.001		<.001		<.001		−3.731		41.337 (2, 61)		<.001
			LCD+exercise		83.6 (10.3)^h^		79.6 (10.0)^c,i^		77.9 (9.9)^c,d,j^		<.001		<.001		.001		−5.662		44.993 (2, 61)		<.001
			TRF+exercise		83.1 (10.4)^h^		80.7 (10.2)^c,i^		79.4 (9.8)^c,d,j^		<.001		<.001		.01		−3.719		18.193 (2, 61)		<.001
			*F* test (*df*)		0.021 (2, 62)		0.058 (2, 62)		0.093 (2, 62)		—		—		—		—		—		—
			*P* value		.98		.94		.91		—		—		—		—		—		—
		**BMI (kg/m^2^)**																			
			TWF+exercise		30.8 (3.2)		30.7 (3.3)		29.5 (3.5)^c,d^		.16		<.001		<.001		−0.981		26.929 (2, 61)		<.001
			LCD+exercise		29.5 (3)		28.7 (3.1)^c^		27.9 (3.2)^c,d^		<.001		<.001		.002		−1.587		19.762 (2, 61)		<.001
			TRF+exercise		30.2 (3)		29.9 (3)		29.2 (2.9)^c,d^		.11		<.001		.005		−1.006		7.225 (2, 61)		.002
			*F* test (*df*)		0.985 (2, 62)		1.977 (2, 62)		1.268 (2, 62)		—		—		—		—		—		—
			*P* value		.38		.14		.29		—		—		—		—		—		—
		**Visceral fat mass**																			
			TWF+exercise		5.92 (2.3)		6.06 (2.4)		5.4 (2.4)^c,d^		.43		.02		<.001		−0.187		6.949 (2, 61)		.002
			LCD+exercise		6.11 (2.3)		5.40 (2.3)^c^		5.08 (2.3)^c^		.009		.001		.22		−1.031		6.110 (2, 61)		.004
			TRF+exercise		6 (2.3)		5.68 (2.5)		5.86 (2.2)		.22		.65		.46		−0.137		0.796 (2, 61)		.46
			*F* test (*df*)		0.040 (2, 62)		0.433 (2, 62)		0.458 (2, 62)		—		—		—		—		—		—
			*P* value		.96		.65		.64		—		—		—		—		—		—
		**Subcutaneous fat mass**																			
			TWF+exercise		31.1 (5.1)^h^		30.9 (5.1)^i^		28.3 (5.9) ^c,d,j^		.63		<.001		<.001		−1.544		8.521 (2,61)		.001
			LCD+exercise		30.8 (6)^h^		28.4 (5.5)^c^		27.2 (5.3)^c^		.001		.001		.22		−3.638		8.912 (2,61)		<.001
			TRF+exercise		30.5 (5.7)		29 (6.7)		29.6 (5.1)^j^		.04		.4		.54		−0.856		2.194 (2,61)		.12
			*F* test (*df*)		0.081 (2,62)		1.278 (2,62)		0.775 (2,62)		—		—		—		—		—		—
			*P* value		.92		.29		.46		—		—		—		—		—		—

^a^MD: mean difference.

^b^TWF: twice-weekly fasting.

^c^Within-group comparisons: indicates *P<*.05 compared to baseline.

^d^Within-group comparisons: indicates *P<*.05 compared to 4 weeks.

^e^LCD: low-calorie diet.

^f^TRF: time-restricted feeding.

^g^Not applicable.

^h^Sex comparisons: indicates *P<*.05 compared to male participant baseline.

^i^Sex comparisons: indicates *P<*.05 compared to 4 weeks in male participants.

^j^Sex comparisons: indicates *P<*.05 compared to 8 weeks in male participants.

### The Interaction Between Sex, Time, and Diet

This study further used linear mixed-effects modeling to analyze the effects of diet (TWF, LCD, and TRF), time (0 wk, 4 wk, and 8 wk), sex (male and female participants), and their interactions on body metrics such as body weight, BMI, visceral fat mass, subcutaneous fat mass, and LBM.

Linear mixed-effects model analyses (Table 5) showed that the sex-diet interaction significantly affected several body composition indicators, validating sex- and diet-specific differences. Time effects on weight (wk 4: hazard ratio [HR]=0.058, wk 8: HR=0.011; *P*<.001) and BMI (wk 4: HR=0.668, wk 8: HR=0.296; *P*<.001) were significant, indicating a decreasing trend over time that was statistically significant (*P*<.001). Female participants lost more weight than male participants in LCD (HR=0.000; *P*<.001), TRF (HR=0.000; *P*<.001), and TWF (HR=0.000; *P*=.001) groups, while male participants had no significant changes in LCD (*P*=.52) and TRF (*P*=.90). Only time effects on BMI were significant. Visceral fat mass had a significant time effect at week 8 (HR=0.62; *P*<.001), with no significant diet sex interaction. Subcutaneous fat mass decreased significantly at week 4 (HR=0.374) and week 8 (HR=0.118; *P*<.001), and female participants in the LCD group had the greatest reduction (HR=85.098; *P*=.02). LBM (week 4: HR=0.558, week 8: HR=0.522; *P*<.001) and muscle mass (week 4: HR=0.580, week 8: HR=0.547; *P*<.001) decreased over time, with female participants having greater decreases (*P*<.001).

**Table 5:** Linear mixed-effects model analysis results for interactions between sex and time.

Index and interaction			HR^a^ (95% CI)		*P* value
**Body weight**					
	LCD^b^	2.35 (0.02-334.13)		.74	
	TRF^c^	3.58 (0.02-769.09)		.64	
	0 wk: female	0 (0.00-0.01)		<.001	
	4 wk: female	0 (0.00-0.00)		<.001	
	8 wk: female	0 (0.00-0.00)		<.001	
	0 wk: male	114.103 (64.00-203.43)		<.001	
	4 wk: male	6.128 (3.44-10.93)		<.001	
**BMI**					
	LCD	0.689 (0.16-2.89)		.61	
	TRF	1.018 (0.22-4.82)		.98	
	0 wk: female	1.616 (0.46-5.68)		.46	
	4 wk: female	1.12 (0.32-3.94)		.86	
	8 wk: female	0.44 (0.13-1.55)		.20	
	0 wk: male	3.103 (2.49-3.87)		<.001	
	4 wk: male	1.991 (1.60-2.48)		<.001	
**Visceral fat mass**					
	LCD	0.946 (0.33-2.74)		.92	
	TRF	1.636 (0.52-5.18)		.40	
	0 wk: female	1.135 (0.44-2.91)		.79	
	4 wk: female	0.947 (0.37-2.43)		.91	
	8 wk: female	0.655 (0.26-1.68)		.38	
	0 wk: male	1.504 (1.19-1.89)		.001	
	4 wk: male	1.313 (1.04-1.65)		.02	
**Subcutaneous fat mass**					
	LCD	0.525 (0.04-6.13)		.61	
	TRF	1.525 (0.11-21.85)		.76	
	0 wk: female	1136.49 (124.91-10,340.63)		<.001	
	4 wk: female	390.099 (42.87-3549.41)		<.001	
	8 wk: female	88.801 (9.76-807.98)		<.001	
	0 wk: male	5.545 (2.73-11.24)		<.001	
	4 wk: male	2.273 (1.12-4.61)		.02	
**Lean body mass**					
	LCD	3.794 (0.59-24.19)		.16	
	TRF	0.962 (0.13-7.16)		.97	
	0 wk: female	0 (0.00-0.00)		<.001	
	4 wk: female	0 (0.00-0.00)		<.001	
	8 wk: female	0 (0.00-0.00)		<.001	
	0Wk: male	3.293 (2.15-5.05)		<.001	
	4 wk: male	1.728 (1.13-2.65)		.01	
**Muscle mass**					
	LCD	3.496 (0.61-19.95)		.16	
	TRF	0.953 (0.14-6.29)		.96	
	0 wk: female	0.000 (0.00-0.00)		<.001	
	4 wk: female	0 (0.00-0.00)		<.001	
	8 wk: female	0 (0.00-0.00)		<.001	
	0Wk: male	3.098 (2.05-4.68)		<.001	
	4 wk: male	1.673 (1.11-2.53)		.02	

^a^HR: hazard ratio.

^b^LCD: low-calorie diet.

^c^TRF: time-restricted feeding.

Linear mixed-effects models showed (Table 6) that the interaction of sex and time significantly affected body composition changes: weight decreased consistently in female (*P*<.001) and increased significantly in male participants at 0 and 4 weeks (*P*<.001). BMI was significantly higher in male participants than in female participants at week 0 (HR=3.103; *P*<.001) and week 4 (HR=1.991; *P*<.001), and in female participants there was a marginal decrease in BMI at week 8 (HR=0.440; *P*=.20). Visceral fat mass increased significantly at week 0 (HR=1.504; *P*=.001) and week 4 (HR=1.313; *P*=.02) in male participants and decreased marginally at 8 weeks in female participants (HR=0.655; *P*=.38). There was a sustained decrease in subcutaneous fat mass in female participants (HR=1136.490 at week 0, HR=390.099 at week 4, and HR=88.801 at week 8; *P*<.001), and a significant increase in male participants at week 0 (HR=5.545; *P*<.001) and week 4 (HR=2.273; *P*=.02).

**Table 6:** Linear mixed-effects model analysis results for interactions between sex and diet.

Index and interaction		HR^a^ (95% CI)	*P* value
**Body weight**			
	4 wk	0.058 (0.039-0.087)	<.001
	8 wk	0.011 (0.007-0.016)	<.001
	LCD^b^: female	0 (0.000-0.001)	<.001
	TRF^c^: female	0 (0.000-0.001)	<.001
	TWF^d^: female	0 (0.000-0.002)	.001
	LCD: male	0.066 (0.000-252)	.52
	TRF: male	0.628 (0.000-1146)	.90
**BMI**			
	4 wk	0.668 (0.572-0.779)	<.001
	8 wk	0.296 (0.254-0.345)	<.001
	LCD: female	0.655 (0.080-5.370)	.69
	TRF: female	0.131 (0.012-1.489)	.10
	TWF: female	0.355 (0.031-4.031)	.40
	LCD: male	0.353 (0.033-3.747)	.39
	TRF: male	0.866 (0.101-7.432)	.90
**Visceral fat mass**			
	4 wk	0.853 (0.727-1.001)	.05
	8 wk	0.619 (0.527-0.726)	<.001
	LCD: female	0.376 (0.078-1.819)	.23
	TRF: female	0.29 (0.047-1.787)	.18
	TWF: female	0.397 (0.064-2.448)	.32
	LCD: male	0.339 (0.058-1.987)	.23
	TRF: male	0.44 (0.088-2.197)	.32
**Subcutaneous fat mass**			
	4 wk	0.374 (0.228-0.614)	<.001
	8 wk	0.118 (0.072-0.193)	<.001
	LCD: female	85.098 (2.233-3243)	.02
	TRF: female	22.783 (0.341-1521)	.15
	TWF: female	56.741 (0.849-3790)	.06
	LCD: male	0.204 (0.003-12.169)	.45
	TRF: male	0.25 (0.006-10.293)	.47
**Lean body mass**			
	4 wk	0.558 (0.411-0.759)	<.001
	8 wk	0.522 (0.384-0.709)	<.001
	LCD: female	0 (0.000-0.000)	<.001
	TRF: female	0 (0.000-0.000)	<.001
	TWF: female	0 (0.000-0.000)	<.001
	LCD: male	1.189 (0.054-26)	.91
	TRF: male	5.34 (0.322-88)	.24
**Muscle mass**			
	4 wk	0.58 (0.432-0.779)	<.001
	8 wk	0.547 (0.407-0.734)	<.001
	LCD: female	0 (0.000-0.000)	<.001
	TRF: female	0 (0.000-0.000)	<.001
	TWF: female	0 (0.000-0.000)	<.001
	LCD: male	1.18 (0.065-21)	.91
	TRF: male	4.943 (0.353-69)	.24

^a^HR: hazard ratio.

^b^LCD: low-calorie diet.

^c^TRF: time-restricted feeding.

^d^TWF: twice-weekly fasting.

## Discussion

### Principal Findings

This study examined the effects of a digital technology intervention on body composition in college students with obesity. Results showed that an intervention program based on smartphone self-monitoring with real-time feedback (adherence >80%, dropout rate only 2.58%) resulted in an average weight loss of 5.05% and significant reductions (*P*<.001) in BMI and weight among participants. All 3 dietary modalities (TRF, TWF, and LCD) combined with exercise were effective in improving body weight, BMI, and adiposity, but there were sex-specific differences: in terms of fat metabolism, male participants showed optimal fat loss in the TWF group, and female participants showed optimal fat loss in the LCD group. Linear mixed models showed that the improvement in subcutaneous fat mass was higher in the LCD group. Regarding muscle maintenance, muscle mass decreased significantly in the TRF group at week 4 (*P*<.001) but stabilized at week 8 (*P*=.91), suggesting a possible muscle-protective effect. The magnitude of muscle loss was significantly higher in female participants than in male participants in all dietary groups. This study revealed that optimal visceral fat mass loss was achieved with a TWF+exercise protocol in male participants with obesity, that female participants with obesity were better suited to the LCD+exercise model, and that TRF excelled in short-term (4-week) muscle protection. These findings provide a new rationale for precision nutritional interventions, confirming that digital technology can be an effective complement to traditional health guidance, particularly in improving behavioral adherence in the young population with obesity.

### Comparison With Previous Work

#### The Impact of Intervention on Muscle Indicators

This study found that male participants in the TRF group showed phase-specific changes in LBM. Literature reviews [4,42,43] indicate that during weight loss, LBM reduction typically accounts for 20% to 30% of total weight loss, with male participants losing 3 times more LBM than female participants [44]. This suggests that LBM is lost during weight reduction, but which dietary method causes the most loss, and when does it occur? One study [45] found that participants on a 4-week TRF regimen experienced significant weight loss (*P*<.05), primarily due to LBM reduction rather than fat loss. A systematic review [46] of 6 observational studies lasting 4 to 8 weeks showed that TRF participants lost more weight but also more LBM, indicating greater short-term LBM reduction with TRF. However, the study by Gabel et al [47] found no significant LBM change after a 12-week 8 hours TRF intervention (*P*>.05), and the study by Kotarsky et al [48] reported no LBM change after 8 weeks of TRF combined with resistance training, suggesting that LBM may stabilize beyond 8 weeks.

Existing preliminary research demonstrates the impact of TRF on LBM during weight loss, but characteristics of the impact over time remain unclear. This study observed a significant LBM decrease in the TRF group after 4 weeks (*P*<.001), representing a potential risk of muscle loss, possibly due to reduced energy intake and muscle breakdown for energy. This is clinically significant, as LBM reduction is linked to health issues like decreased metabolic rate, reduced insulin sensitivity, and increased osteoporosis risk. By week 8, LBM tended to stabilize, with a slight increase in the female group, suggesting that TRF combined with exercise may positively maintain or increase LBM in the long term. Combining resistance training and optimizing nutritional intake (eg, ensuring adequate protein and nutrient distribution within the eating window) may mitigate muscle loss during TRF. However, more long-term randomized controlled trials and metabolic studies are needed to clarify mechanisms and evaluate intervention strategies, especially personalized approaches for populations with obesity, to minimize muscle loss and improve long-term health outcomes.

#### The Impact of Intervention on Fat Indicators

This study found that different dietary approaches combined with exercise exhibited sex specificity in fat indices. Visceral fat mass is an important and independent risk factor for cardiovascular metabolic diseases [49], and both total fat mass and visceral fat mass accumulation are closely associated with the occurrence of cardiovascular diseases, stroke, hypertension, and insulin resistance [50-52]. Effective interventions to reduce visceral fat mass in individuals with obesity may improve health outcomes. Previous systematic reviews, meta-analyses, and randomized experiments have confirmed [53-56] that combined exercise and intermittent fasting interventions can effectively reduce body fat and visceral fat mass in adults with obesity; a 6-month exercise combined with an LCD intervention in female participants with obesity found that visceral fat mass (*P*=.03) and body fat percentage (*P*=.04) significantly decreased after the intervention [57]. These studies demonstrate the impact of exercise combined with diet on fat indices, but no sex specificity was found in different dietary approaches. This study found that in the 3 dietary approaches, male participants in the TWF+exercise group showed a greater reduction in fat indices, while female participants in the LCD+exercise group showed a more significant decrease in fat indices. The trial by Trouwborst et al [35] followed an 8-week LCD (800 kcal/day) and found that after weight loss, fat reduction was more pronounced in male participants than in female participants. After weight loss, the changes in visceral or abdominal fat in male participants were more pronounced, while subcutaneous fat mass in female participants decreased more significantly, which is opposite to the results of this study. This discrepancy may be due to the intervention protocol or measurement methods.

This study showed that the combination of the 3 dietary approaches with exercise has a significant effect on fat indices. This is consistent with the vast majority of studies, but differences were found through mean comparisons. Male participants showed better results with the TWF diet combined with exercise, while female participants showed better results with the LCD diet combined with exercise, especially in visceral fat mass. Therefore, it is suggested that male participants may lean toward the TWF diet combined with exercise during weight loss, and female participants may lean toward the LCD diet combined with exercise.

#### Comprehensive Effects of Interventions on Body Composition: Analysis Based on the Interaction of Diet, Time, and Sex

This study further revealed the interaction effects of diet, time, and sex on body composition through a linear mixed model. For instance, in the analysis of body weight, the significant interaction between time and sex indicated that the impact of intervention duration on weight varied by sex, which may be related to biological differences in fat metabolism and hormone levels between male participants and female participants [58]. Specifically, the weight of the female group decreased over time, while the male group experienced weight gain at certain time points, suggesting the need for sex-specific personalized weight management strategies. Regarding dietary factors, the slight positive effect of TRF on body weight and visceral fat mass warrants further investigation into its caloric and nutritional composition.

In addition, in other aspects of body composition, such as LBM and muscle mass, the significant interaction between time and sex reflected that the intervention’s effect on lean tissue exhibited sex specificity, which is associated with differences in muscle composition and exercise responsiveness between male participants and female participants [59]. These findings provide a basis for precise diet intervention, emphasizing the importance of incorporating the sex dimension into health management and dynamically adjusting strategies. Future research can further integrate biochemical indicators and genetic data, explore the molecular mechanism of the interaction between sex, diet, and time, and thus optimize personalized intervention strategies.

In terms of the time dimension, the significant effects of 4 and 8 weeks on body weight, BMI, and subcutaneous fat mass indicated that prolonged intervention duration effectively improved weight and fat-related indicators, supporting the necessity of long-term interventions. In the interaction between diet and sex, the significant effects of “LCD: female,” “TRF: female,” and “TWF: female” on body weight suggested that specific dietary approaches may offer greater advantages for weight management in the female population, potentially due to differences in metabolic characteristics and hormone levels [60-62]. For example, the significant positive effect of LCD: female on subcutaneous fat mass may reflect a unique regulatory mechanism of this dietary pattern on subcutaneous fat mass in female participants, which requires further investigation into its nutritional composition.

#### Self-Selection of Dietary Regimens: Coexistence of Selection Bias and Practical Application Advantages

In this study, the voluntary selection of intervention groups by participants may have introduced selection bias, which could affect the validity and generalizability of the results. Self-selection bias often arises when participants choose intervention groups based on personal preferences, beliefs, or expectations, potentially leading to baseline differences between groups and confounding the interpretation of intervention effects. Although the analysis showed no significant differences between groups at baseline, underlying differences in motivation, adherence, or health status may still influence the results. In addition, the lack of blinding of participants may lead to performance bias and measurement bias. For example, participants may alter their behavior or report outcomes differently based on their knowledge of the intervention group. Such biases are common in nonblinded studies and may compromise the objectivity of the results. However, a potential advantage of self-selection is that it may improve participant adherence. Studies have shown that designs that allow participants to choose diets are more reflective of real-world implementations [63]. In addition, self-selection of dietary approaches may enhance adherence, making it easier for participants to sustain their chosen regimen and achieve ideal weight loss outcomes. For example, a study on patients with type 2 diabetes demonstrated that allowing patients to choose their diets based on previous experience and dietary guidance exhibited practical applicability and effectiveness in managing type 2 diabetes [64].

Therefore, although self-selection and the lack of blinding may introduce bias, their positive impact on adherence is also worth noting. Future research should adopt randomized and blinded designs by balancing the trade-offs between bias and adherence, thereby enhancing the reliability of the study.

The initial phase of weight loss plays a crucial role in shaping future weight management. Although an 8-week intervention is insufficient to evaluate long-term maintenance effects, it provides important insights into short- to medium-term outcomes, which are fundamental and critical for the prevention and control of obesity. This is supported by existing literature. For instance, a recent meta-review [65] highlighted that among systematic reviews (9/17, 53%) and meta-analyses (8/17, 47%) focusing on PA and PA+diet applications, the most common intervention durations were 6 weeks (3/17, 18%) and 8 weeks (3/17, 18%). Specifically, for studies involving PA+diet applications, the most common duration was 8 weeks (3/12, 25%). This further validates the suitability of our 8-week design in obesity-related intervention research.

In addition, the relationship between the effects of short-term interventions and long-term weight management has been explored in other studies. For example, one study [66] suggests that short-term body weight variability has potential value in predicting long-term weight changes. Combining these findings, short-term interventions (such as 8 weeks) not only provide significant insights into short- to medium-term outcomes but also lay the foundation for long-term weight management. Although the 8-week intervention period is relatively short, it aligns with the common durations found in the literature and holds a certain reference value in predicting long-term weight changes. Therefore, in obesity prevention and control intervention research, the 8-week design is both reasonable and applicable, while long-term monitoring and intervention strategies should also be integrated to achieve more sustainable weight management outcomes.

### Study Strengths and Limitations

This study has the following strengths. First, it innovatively integrated digital technology into an obesity intervention and the enhancement of behavioral adherence through self-monitoring with feedback. Second, the combination of 3 dietary patterns (TWF, LCD, and TRF) with a standardized exercise regimen enables a direct comparison of intervention effects. Third, this study specifically explored the differential response of male and female participants to different dietary interventions, laying the foundation for personalized weight management strategies. Fourth, the digital platform of this study achieved a high level of adherence rate and a low dropout rate, validating the feasibility of technology-driven interventions in real-world scenarios. Finally, this study covers body composition metrics (muscle mass and visceral or subcutaneous fat mass) and longitudinal data analysis, providing dual insights into short-term and long-term effects.

Nevertheless, the following shortcomings need to be noted: first, self-selection could lead to baseline differences between groups (eg, health awareness, motivation, or baseline health status), which may affect the interpretation of the results. In addition, participants might adjust their behavior based on their knowledge of the intervention or be influenced by subjective expectations when reporting outcomes, potentially introducing performance and measurement biases. Furthermore, self-selection may be associated with other health behaviors (eg, exercise or sleep) that could independently influence the results. Future randomized controlled trials are needed to validate the findings. Second, the sample was limited to the youth population, which limits the applicability of the results to the older adult population or clinical scenarios. Third, the 8-week intervention cycle does not allow for the extrapolation of the sustainability of long-term effects. Fourth, potential confounders such as psychological factors (eg, stress and motivation) and lifestyle variables (eg, sleep quality) were not assessed, which may affect the interpretation of the results.

### Conclusions

This study demonstrated that diet and exercise interventions augmented by digital technology were effective in improving body composition in college students with obesity and differed by sex. For example, male students significantly reduced visceral fat mass with TWF+exercise. Female students lost more subcutaneous fat mass with LCD+exercise. In addition, self-monitoring with feedback significantly improved adherence, reduced participant burden, and enhanced intervention effects through real-time feedback mechanisms, thus validating the effectiveness of the closed-loop monitoring-feedback behavior modification and highlighting its role as a scalable tool for obesity management. Sex and diet interactions had a significant effect on subcutaneous fat mass, with an LCD being useful in female students and TRF needing to be combined with protein supplementation. Taken together, these findings suggest that digital technology has great potential for obesity management, highlighting the importance of incorporating sex-specific factors into weight loss strategies, and showing that future research should explore long-term outcomes, older adult populations, and personalized nutrition-exercise prescriptions.

## Appendix

Multimedia Appendix 1 Specific intervention methods (knowledge, exercise, diet, self-monitoring, and feedback).

Multimedia Appendix 2 Intervention protocol.

Multimedia Appendix 3 Comparison of compliance by dietary patterns and sex during the intervention period.

Multimedia Appendix 4 Trend charts of changes in fat indicators and muscle indicators.

## Abbreviations

HR: hazard ratio
LBM: lean body mass
LCD: low-calorie diet
MD: mean difference
PA: physical activity
TRF: time-restricted feeding
TWF: twice-weekly fasting

## Data Availability

The datasets generated or analyzed during this study are not publicly available due to the need to protect the privacy and information of the study participants but are available from the corresponding author on reasonable request.

## References

[1] Romieu I, Dossus L, Barquera S, Blottière HM, Franks PW, Gunter M, Hwalla N, Hursting SD, Leitzmann M, Margetts B, Nishida C, Potischman N, Seidell J, Stepien M, Wang Y, Westerterp K, Winichagoon P, Wiseman M, Willett WC (2017). Energy balance and obesity: what are the main drivers?. Cancer Causes Control.

[2] Cheung K, Chan V, Chan S, Wong MM, Chung GK, Cheng WY, Lo K, Zeng F (2024). Effect of intermittent fasting on cardiometabolic health in the chinese population: a meta-analysis of randomized controlled trials. Nutrients.

[3] Sanchez-Lastra MA, Ding D, Del Pozo Cruz B, Dalene KE, Ayán C, Ekelund U, Tarp J (2024). Joint associations of device-measured physical activity and abdominal obesity with incident cardiovascular disease: a prospective cohort study. Br J Sports Med.

[4] Cava E, Yeat NC, Mittendorfer B (2017). Preserving healthy muscle during weight loss. Adv Nutr.

[5] Aras M, Tchang BG, Pape J (2021). Obesity and diabetes. Nurs Clin North Am.

[6] Gallagher EJ, LeRoith D (2015). Obesity and diabetes: the increased risk of cancer and cancer-related mortality. Physiol Rev.

[7] Moghaddam AA, Woodward M, Huxley R (2007). Obesity and risk of colorectal cancer: a meta-analysis of 31 studies with 70,000 events. Cancer Epidemiol Biomarkers Prev.

[8] Wolin KY, Carson K, Colditz GA (2010). Obesity and cancer. Oncologist.

[9] (2023). Obesity: Identification, Assessment and Management. National Institute for Health and Care Excellence.

[10] Sarwer DB, von Sydow Green A, Vetter ML, Wadden TA (2009). Behavior therapy for obesity: where are we now?. Curr Opin Endocrinol Diabetes Obes.

[11] Pappa GL, Cunha TO, Bicalho PV, Ribeiro A, Couto Silva AP, Meira W Jr, Beleigoli AM (2017). Factors associated with weight change in online weight management communities: a case study in the LoseIt Reddit Community. J Med Internet Res.

[12] Sforzo GA, Moore M, Moore GE, Harenberg S (2021). Comment on "health coaching: 100 strategies for weight loss: a systematic review and meta-analysis". Adv Nutr.

[13] (2019). WHO guideline: recommendations on digital interventions for health system strengthening. World Health Organization.

[14] Mateo-Orcajada A, Ponce-Ramírez CM, Abenza-Cano L, Vaquero-Cristóbal R (2024). Effects of 10 weeks of walking with mobile step-tracking apps on body composition, fitness, and psychological state in adolescents who are overweight and obese: randomized controlled trial. J Med Internet Res.

[15] Li M, Liu S, Yu B, Li N, Lyu A, Yang H, He H, Zhang N, Ma J, Sun M, Du H, Gao R (2025). Assessing the effectiveness of digital health behavior strategies on type 2 diabetes management: systematic review and network meta-analysis. J Med Internet Res.

[16] Palacz-Poborczyk I, Idziak P, Januszewicz A, Luszczynska A, Quested E, Naughton F, Hagger MS, Pagoto S, Verboon P, Robinson S, Kwasnicka D (2022). Developing the "choosing health" digital weight loss and maintenance intervention: intervention mapping study. J Med Internet Res.

[17] Hendrie GA, Baird DL, James-Martin G, Brindal E, Brooker PG (2025). Weight loss patterns and outcomes over 12 months on a commercial weight management program (CSIRO total wellbeing diet online): large-community cohort evaluation study. J Med Internet Res.

[18] Taylor K, Indulkar T, Thompson B, Pinkard C, Barron E, Frost T, Jayawardane P, Davies N, Bakhai C, Forouhi NG, Aveyard P, Jebb S, Valabhji J (2024). Early outcomes of referrals to the English National Health Service Digital Weight Management Programme. Obesity (Silver Spring).

[19] Wang Y, Min J, Khuri J, Xue H, Xie B, A Kaminsky L, J Cheskin L (2020). Effectiveness of mobile health interventions on diabetes and obesity treatment and management: systematic review of systematic reviews. JMIR Mhealth Uhealth.

[20] Klasnja P, Pratt W (2012). Healthcare in the pocket: mapping the space of mobile-phone health interventions. J Biomed Inform.

[21] Lugones-Sanchez C, Recio-Rodriguez JI, Agudo-Conde C, Repiso-Gento I, G Adalia E, Ramirez-Manent JI, Sanchez-Calavera MA, Rodriguez-Sanchez E, Gomez-Marcos MA, Garcia-Ortiz L (2022). Long-term effectiveness of a smartphone app combined with a smart band on weight loss, physical activity, and caloric intake in a population with overweight and obesity (evident 3 study): randomized controlled trial. J Med Internet Res.

[22] Chew HS, Koh WL, Ng JS, Tan KK (2022). Sustainability of weight loss through smartphone apps: systematic review and meta-analysis on anthropometric, metabolic, and dietary outcomes. J Med Internet Res.

[23] Tully L, Sorensen J, O'Malley G (2021). Pediatric weight management through mHealth compared to face-to-face care: cost analysis of a randomized control trial. JMIR Mhealth Uhealth.

[24] Burke LE, Wang J, Sevick MA (2011). Self-monitoring in weight loss: a systematic review of the literature. J Am Diet Assoc.

[25] Chatterjee A, Prinz A, Gerdes M, Martinez S (2021). Digital interventions on healthy lifestyle management: systematic review. J Med Internet Res.

[26] Burke LE, Choo J, Music E, Warziski M, Styn MA, Kim Y, Sevick MA (2006). PREFER study: a randomized clinical trial testing treatment preference and two dietary options in behavioral weight management--rationale, design and baseline characteristics. Contemp Clin Trials.

[27] Anton S, Das SK, McLaren C, Roberts SB (2021). Application of social cognitive theory in weight management: time for a biological component?. Obesity (Silver Spring).

[28] Johnson H, Huang D, Liu V, Ammouri MA, Jacobs C, El-Osta A (2025). Impact of digital engagement on weight loss outcomes in obesity management among individuals using GLP-1 and dual GLP-1/GIP receptor agonist therapy: retrospective cohort service evaluation study. J Med Internet Res.

[29] Arnott B, Kitchen CE, Ekers D, Gega L, Tiffin PA (2020). Behavioural activation for overweight and obese adolescents with low mood delivered in a community setting: feasibility study. BMJ Paediatr Open.

[30] Burke LE, Sereika SM, Bizhanova Z, Parmanto B, Kariuki J, Cheng J, Beatrice B, Cedillo M, Pulantara IW, Wang Y, Loar I, Conroy MB (2022). The effect of tailored, daily, smartphone feedback to lifestyle self-monitoring on weight loss at 12 months: the SMARTER randomized clinical trial. J Med Internet Res.

[31] Allegra S, Chiara F, Di Grazia D, Gaspari M, De Francia S (2023). Evaluation of sex differences in preclinical pharmacology research: how far is left to go?. Pharmaceuticals (Basel).

[32] Garcia-Sifuentes Y, Maney DL (2021). Reporting and misreporting of sex differences in the biological sciences. Elife.

[33] Cooper AJ, Gupta SR, Moustafa AF, Chao AM (2021). Sex/gender differences in obesity prevalence, comorbidities, and treatment. Curr Obes Rep.

[34] Liqiang S, Fang-Hui L, Minghui Q, Haichun C (2023). Threshold effect and sex characteristics of the relationship between chronic inflammation and BMI. BMC Endocr Disord.

[35] Trouwborst I, Goossens GH, Astrup A, Saris WH, Blaak EE (2021). Sexual dimorphism in body weight loss, improvements in cardiometabolic risk factors and maintenance of beneficial effects 6 months after a low-calorie diet: results from the randomized controlled DiOGenes trial. Nutrients.

[36] Sun ML, Yao W, Wang XY, Gao S, Varady KA, Forslund SK, Zhang M, Shi ZY, Cao F, Zou BJ, Sun MH, Liu KX, Bao Q, Xu J, Qin X, Xiao Q, Wu L, Zhao YH, Zhang DY, Wu QJ, Gong TT (2024). Intermittent fasting and health outcomes: an umbrella review of systematic reviews and meta-analyses of randomised controlled trials. EClinicalMedicine.

[37] Sampieri A, Paoli A, Spinello G, Santinello E, Moro T (2024). Impact of daily fasting duration on body composition and cardiometabolic risk factors during a time-restricted eating protocol: a randomized controlled trial. J Transl Med.

[38] Vázquez-Lorente H, Herrera-Quintana L, Molina-López J, López-González B, Planells E (2023). Sociodemographic, anthropometric, body composition, nutritional, and biochemical factors influenced by age in a postmenopausal population: a cross-sectional study. Metabolites.

[39] Haas K, Hayoz S, Maurer-Wiesner S (2019). Effectiveness and feasibility of a remote lifestyle intervention by dietitians for overweight and obese adults: pilot study. JMIR Mhealth Uhealth.

[40] Björnsdottir S, Ulfsdottir H, Gudmundsson EF, Sveinsdottir K, Isberg AP, Dobies B, Akerlie Magnusdottir GE, Gunnarsdottir T, Karlsdottir T, Bjornsdottir G, Sigurdsson S, Oddsson S, Gudnason V (2024). User engagement, acceptability, and clinical markers in a digital health program for nonalcoholic fatty liver disease: prospective, single-arm feasibility study. JMIR Cardio.

[41] Dunn CG, Turner-McGrievy GM, Wilcox S, Hutto B (2019). Dietary self-monitoring through calorie tracking but not through a digital photography app is associated with significant weight loss: the 2SMART pilot study-a 6-month randomized trial. J Acad Nutr Diet.

[42] Magkos F, Fraterrigo G, Yoshino J, Luecking C, Kirbach K, Kelly SC, de las Fuentes L, He S, Okunade AL, Patterson BW, Klein S (2016). Effects of moderate and subsequent progressive weight loss on metabolic function and adipose tissue biology in humans with obesity. Cell Metab.

[43] Verreijen AM, Verlaan S, Engberink MF, Swinkels S, de Vogel-van den Bosch J, Weijs PJ (2015). A high whey protein-, leucine-, and vitamin D-enriched supplement preserves muscle mass during intentional weight loss in obese older adults: a double-blind randomized controlled trial. Am J Clin Nutr.

[44] Millward DJ, Truby H, Fox KR, Livingstone MB, Macdonald IA, Tothill P (2013). Sex differences in the composition of weight gain and loss in overweight and obese adults. Br J Nutr.

[45] Chen JH, Lu LW, Ge Q, Feng D, Yu J, Liu B, Zhang R, Zhang X, Ouyang C, Chen F (2023). Missing puzzle pieces of time-restricted-eating (TRE) as a long-term weight-loss strategy in overweight and obese people? A systematic review and meta-analysis of randomized controlled trials. Crit Rev Food Sci Nutr.

[46] Pellegrini M, Cioffi I, Evangelista A, Ponzo V, Goitre I, Ciccone G, Ghigo E, Bo S (2020). Effects of time-restricted feeding on body weight and metabolism. A systematic review and meta-analysis. Rev Endocr Metab Disord.

[47] Gabel K, Hoddy KK, Haggerty N, Song J, Kroeger CM, Trepanowski JF, Panda S, Varady KA (2018). Effects of 8-hour time restricted feeding on body weight and metabolic disease risk factors in obese adults: a pilot study. Nutr Health Aging.

[48] Kotarsky CJ, Johnson NR, Mahoney SJ, Mitchell SL, Schimek RL, Stastny SN, Hackney KJ (2021). Time-restricted eating and concurrent exercise training reduces fat mass and increases lean mass in overweight and obese adults. Physiol Rep.

[49] Khalafi M, Malandish A, Rosenkranz SK, Ravasi AA (2021). Effect of resistance training with and without caloric restriction on visceral fat: a systemic review and meta-analysis. Obes Rev.

[50] Karpe F, Pinnick KE (2015). Biology of upper-body and lower-body adipose tissue--link to whole-body phenotypes. Nat Rev Endocrinol.

[51] Link JC, Reue K (2017). Genetic basis for sex differences in obesity and lipid metabolism. Annu Rev Nutr.

[52] Pulit SL, Karaderi T, Lindgren CM (2017). Sexual dimorphisms in genetic loci linked to body fat distribution. Biosci Rep.

[53] Kazeminasab F, Baharlooie M, Karimi B, Mokhtari K, Rosenkranz SK, Santos HO (2024). Effects of intermittent fasting combined with physical exercise on cardiometabolic outcomes: systematic review and meta-analysis of clinical studies. Nutr Rev.

[54] Byrne NM, Sainsbury A, King NA, Hills AP, Wood RE (2018). Intermittent energy restriction improves weight loss efficiency in obese men: the MATADOR study. Int J Obes (Lond).

[55] Carter S, Clifton PM, Keogh JB (2018). Effect of intermittent compared with continuous energy restricted diet on glycemic control in patients with type 2 diabetes: a randomized noninferiority trial. JAMA Netw Open.

[56] Khalafi M, Maleki AH, Ehsanifar M, Symonds ME, Rosenkranz SK (2025). Longer-term effects of intermittent fasting on body composition and cardiometabolic health in adults with overweight and obesity: a systematic review and meta-analysis. Obes Rev.

[57] Zalejska-Fiolka J, Birková A, Wielkoszyński T, Hubková B, Szlachta B, Fiolka R, Błaszczyk U, Kuzan A, Gamian A, Mareková M, Toborek M (2022). Loss of skeletal muscle mass and intracellular water as undesired outcomes of weight reduction in obese hyperglycemic women: a short-term longitudinal study. Int J Environ Res Public Health.

[58] Palmisano BT, Zhu L, Eckel RH, Stafford JM (2018). Sex differences in lipid and lipoprotein metabolism. Mol Metab.

[59] Hill EC, Housh TJ, Smith CM, Schmidt RJ, Johnson GO (2018). Gender- and muscle-specific responses during fatiguing exercise. J Strength Cond Res.

[60] Kang J, Shi X, Fu J, Li H, Ma E, Chen W (2022). Effects of an intermittent fasting 5:2 plus program on body weight in Chinese adults with overweight or obesity: a pilot study. Nutrients.

[61] de Oliveira Maranhão Pureza IR, da Silva Junior AE, Silva Praxedes DR, Lessa Vasconcelos LG, de Lima Macena M, Vieira de Melo IS, de Menezes Toledo Florêncio TM, Bueno NB (2021). Effects of time-restricted feeding on body weight, body composition and vital signs in low-income women with obesity: a 12-month randomized clinical trial. Clin Nutr.

[62] Pearl RL, Wadden TA, Tronieri JS, Berkowitz RI, Chao AM, Alamuddin N, Leonard SM, Carvajal R, Bakizada ZM, Pinkasavage E, Gruber KA, Walsh OA, Alfaris N (2018). Short- and long-term changes in health-related quality of life with weight loss: results from a randomized controlled trial. Obesity (Silver Spring).

[63] Jospe MR, Roy M, Brown RC, Haszard JJ, Meredith-Jones K, Fangupo LJ, Osborne H, Fleming EA, Taylor RW (2020). Intermittent fasting, Paleolithic, or Mediterranean diets in the real world: exploratory secondary analyses of a weight-loss trial that included choice of diet and exercise. Am J Clin Nutr.

[64] Alzahrani AH, Skytte MJ, Samkani A, Thomsen MN, Astrup A, Ritz C, Chabanova E, Frystyk J, Holst JJ, Thomsen HS, Madsbad S, Haugaard SB, Krarup T, Larsen TM, Magkos F (2021). Body weight and metabolic risk factors in patients with type 2 diabetes on a self-selected high-protein low-carbohydrate diet. Eur J Nutr.

[65] Bushey E, Wu Y, Wright A, Pescatello L (2024). The influence of physical activity and diet mobile apps on cardiovascular disease risk factors: meta-review. J Med Internet Res.

[66] Turicchi J, O'Driscoll R, Lowe M, Finlayson G, Palmeira AL, Larsen SC, Heitmann BL, Stubbs J (2021). The impact of early body-weight variability on long-term weight maintenance: exploratory results from the NoHoW weight-loss maintenance intervention. Int J Obes (Lond).

