# Supplementary material for: The Impact of Digital Technology–Based Exercise Combined With Dietary Intervention on Body Composition in College Students With Obesity: Prospective Study
Source: J Med Internet Res. 2025 Jun 2;27:e65868. doi: 10.2196/65868 (PMC12171640; doi:10.2196/65868)
Supplement: Multimedia Appendix 1 [file jmir_v27i1e65868_app1.pdf]

S1:Knowledge, exercise, diet, self-monitoring, and feedback intervention are the specific descriptions of this passage.

Knowledge intervention

During the baseline phase, all participants attended a face-to-face intervention course. Subsequently, the research team demonstrated the use of the WeChat mini-program, including how to monitor diet, physical activity (PA), fill out questionnaires, and set up a WeChat group. Additionally, each week, professionals created PowerPoint presentations covering topics such as prevention of sports injuries, the 5+2 intermittent fasting method, the 16/8 time-restricted eating pattern, low-calorie dieting, the weight loss cycle (from weight reduction to weight maintenance), the correct posture for running, exercise principles, the key role of diet during fat loss, and health knowledge for weight maintenance. The research team shared these PowerPoint presentations within the group for self-study by the participants and required them to submit their learning notes online.

Exercise intervention

The exercise intervention consists of two one-hour intensive training sessions per week, guided by professional coaches. The training regimen is high-intensity interval training, focusing primarily on cardiovascular endurance and strength exercises. Additionally, there is a requirement for at least five sessions of sunlight running per week, each not less than 2 km in distance. All of these activities are recorded and uploaded to the application.

Diet intervention

A low-calorie diet (LCD) refers to a dietary regimen with a total daily caloric intake of 800 kcal [35]. Intermittent fasting (IF) includes alternate-day fasting (ADF), the twice-per week fasting diet (TWF), and time-restricted feeding (TRF). Given that ADF requires multiple fasting days per week and considering the academic burden of university students, this diet method was excluded from the study.TWF typically involves fasting for 2 days per week, consuming no more than 600 kcal per fasting day, and unrestricted eating for the other 5 days. TRF entails a daily fasting period of 12 to 24 hours, with the remaining time allocated for eating; in this study, we selected a 16-hour fasting period and an 8-hour window for unrestricted eating [36,37]. In this study, participants were allowed to freely choose their diet method, which helped to enhance their compliance.

Monitoring

The WeChat mini-program was primarily used for self-monitoring, where participants could provide feedback on their weekly diet, exercise, and survey responses via their personal smartphones. Dietary feedback included meal intake, recorded once daily; exercise feedback was fixed at twice a week for one hour of exercise each time, including the "Sunshine Run"; the questionnaire was conducted once a week, aiming to help the research team understand the participants' status in order to better tailor feedback to their individual needs.

Feedback

The WeChat group was primarily utilized by the research team to analyze the information uploaded by participants and to provide them with feedback. This feedback comprised a weekly educational session on knowledge, exercise completion, dietary intake, and psychological motivation. The knowledge session covered the principles and methods of the three dietary approaches, knowledge about fat reduction cycles, exercise methods and principles, and prevention of sports injuries. Exercise feedback was based on the analysis of the exercise check-in data submitted by the participants. The research team published the analysis results within the group at 22:00 every day and offered commendations to those who consistently engaged in exercise. Dietary feedback was provided based on the content and images of the participants' daily food intake that were uploaded. For participants who did not meet the dietary standards, the research team issued prompts within the group. In terms of psychological motivation, the research team sent encouraging messages in the WeChat group every day, such as: "Do not back down in the face of difficulties; stand tall, advance bravely, ignite your passion, and shed your sweat. Let fat reduction light up your healthy life. Keep it up!"
