# Supplementary material for: The Impact of Digital Technology–Based Exercise Combined With Dietary Intervention on Body Composition in College Students With Obesity: Prospective Study
Source: J Med Internet Res. 2025 Jun 2;27:e65868. doi: 10.2196/65868 (PMC12171640; doi:10.2196/65868)
Supplement: Multimedia Appendix 2 [file jmir_v27i1e65868_app2.pdf]

S2:Intervention Protocol

<table>
<tr><td colspan="2"></td><td>1Week</td><td>2Week</td><td>3Week</td><td>4Week</td><td>5Week</td><td>6Week</td><td>7Week</td><td>8Week</td></tr>
<tr><td colspan="2">Knowledge Program</td><td>1. In-person intervention course.
2. Demonstration of Wechat mini-program usage and group creation.
3. Delivery of knowledge on the three dietary approaches, with content distributed within the group.</td><td>Creation of educational materials on exercise-related injuries based on participant feedback.</td><td>Creation of educational materials on fat loss cycles based on participant feedback.</td><td>Creation of educational materials on proper running technique based on participant feedback.</td><td>Creation of educational materials on exercise principles based on participant feedback.</td><td>Creation of educational materials on the role of diet based on participant feedback.</td><td>Creation of educational materials on maintaining healthy weight based on participant feedback.</td><td>Answering participants' questions.</td></tr>
<tr><td rowspan="4">Exercise Program</td><td>Training Content</td><td>Two training sessions per week (adaptive training, primarily games).</td><td colspan="7">1. Two training sessions per week (high-intensity interval training, focusing on cardiorespiratory endurance and strength exercises).
2. At least five runs per week, each at least 2KM.</td></tr>
<tr><td>Training Time and Location</td><td>Monday to Thursday, 17:30-18:30 at the Shell Stadium.</td><td colspan="7">1.Every Monday to Thursday, 17:30-18:30 PM, training at Yaohu Stadium, Yaohu Campus of Jiangxi Normal University, Nanchang, China
2.Sunshine runs are untimed and must be self-reported by participants; location options are the Long Sheng Stadium or the Shell Stadium.</td></tr>
<tr><td>Training Intensity</td><td>60%～70%HR_{max}</td><td colspan="7">65%～75%HRmax</td></tr>
<tr><td>Warm-up and Cool-down Activities</td><td colspan="8">Each training session includes a 5-minute warm-up at 60% HRmax and a 5-minute cool-down at 60% HRmax.</td></tr>
<tr><td rowspan="3">Dietary Plan</td><td>TWF[36]</td><td colspan="8">Fasting for two days per week, consuming 300kcal each day, with normal diet on the remaining five days.</td></tr>
<tr><td>LCD[35]</td><td colspan="8">Daily caloric intake not exceeding 800kcal.</td></tr>
<tr><td>TRF[37]</td><td colspan="8">Daily caloric intake not exceeding 800kcal.</td></tr>
<tr><td rowspan="3">Self-Monitoring Scheme</td><td>Diet</td><td colspan="8">Upload daily diet content and images, once daily.</td></tr>
<tr><td>Exercise</td><td colspan="8">Upload participation in weekly training sessions and sunshine runs, as well as the distance covered, once weekly.</td></tr>
<tr><td>Questionnaire Survey</td><td colspan="8">Include self-reported hunger levels, hunger tolerance, the number of physical activities over 20 minutes per week, sitting duration per week, challenges, and confusions, once weekly.</td></tr>
<tr><td rowspan="4">Feedback Scheme</td><td>Participant Confusion and Challenges</td><td colspan="8">For participants' confusion and challenges, the research team will provide answers through WeChat or during training sessions and create corresponding knowledge explanations to address these issues.</td></tr>
<tr><td>Diet</td><td colspan="8">Research team will provide feedback in the group for participants not meeting dietary standards based on daily uploaded content and images.</td></tr>
<tr><td>Exercise</td><td colspan="8">Analysis of exercise check-in data uploaded by participants, with results announced in the group at 22:00 daily and praise for consistent exercisers.</td></tr>
<tr><td>Motivational Messages</td><td colspan="8">The research team will send motivational messages daily in the WeChat group, such as: "Don't retreat due to difficulties; stand tall, move forward bravely, ignite your passion, sweat it out, let fat loss ignite your healthy life, keep going!"</td></tr>
</table>

TWF: twice-per week fasting diet, LCD::low-calorie diet, TRF: time-restricted feeding
