# Supplementary material for: The Impact of Digital Technology–Based Exercise Combined With Dietary Intervention on Body Composition in College Students With Obesity: Prospective Study
Source: J Med Internet Res. 2025 Jun 2;27:e65868. doi: 10.2196/65868 (PMC12171640; doi:10.2196/65868)
Supplement: Multimedia Appendix 3 [file jmir_v27i1e65868_app3.pdf]

Table S3:Comparison of Compliance by Dietary Patterns and Gender During the Intervention Period

This study analyzed the effects of gender and dietary patterns on exercise and dietary compliance. The results showed no significant differences between genders: males had slightly higher exercise compliance (83.88% vs. 81.88%), while females had slightly higher dietary compliance (84.38% vs. 82.63%). There were also no significant differences among dietary patterns, with time-restricted eating (TRF) showing the highest exercise compliance (84.50%) and low-carbohydrate diet (LCD) showing the highest dietary compliance (85.00%). Overall, compliance rates in all groups were relatively high (all exceeding 80%), indicating good adherence to exercise and dietary requirements among participants.

Table 6 Differences in Compliance by Gender and Dietary Patterns

	Exercise Compliance Score Mean (SD)	Exercise Compliance%	Dietary Compliance Score Mean (SD)	Dietary Compliance%
Gender	6.62(1.30)	82.75%	6.69(1.15)	83.63%
Male	6.71(1.11)	83.88%	6.61(1.31)	82.63%
Female	6.55(1.45)	81.88%	6.75(0.97)	84.38%
P	.491		.480	
Dietary Patterns	6.69(1.15)	83.63%	6.69(1.15)	83.63%
TWF	6.75(1.32)	84.38%	6.71(1.06)	83.88%
LCD	6.41(1.41)	80.13%	6.80(1.24)	85.00%
TRF	6.76(1.07)	84.50%	6.48(1.15)	81.00%
P	.494		.386	
