# Supplementary material for: The Impact of Digital Technology–Based Exercise Combined With Dietary Intervention on Body Composition in College Students With Obesity: Prospective Study
Source: J Med Internet Res. 2025 Jun 2;27:e65868. doi: 10.2196/65868 (PMC12171640; doi:10.2196/65868)

Figure S4 :Graph of the trends of three groups of muscle and fat indicators over time

Figure 2 Graph of the trends of three groups of muscle indicators over time

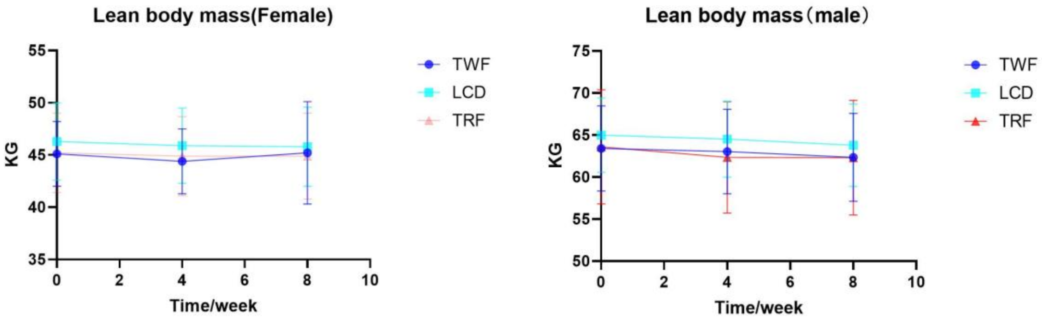

Figure 3 Graph of the trends of three groups of fat indicators over time

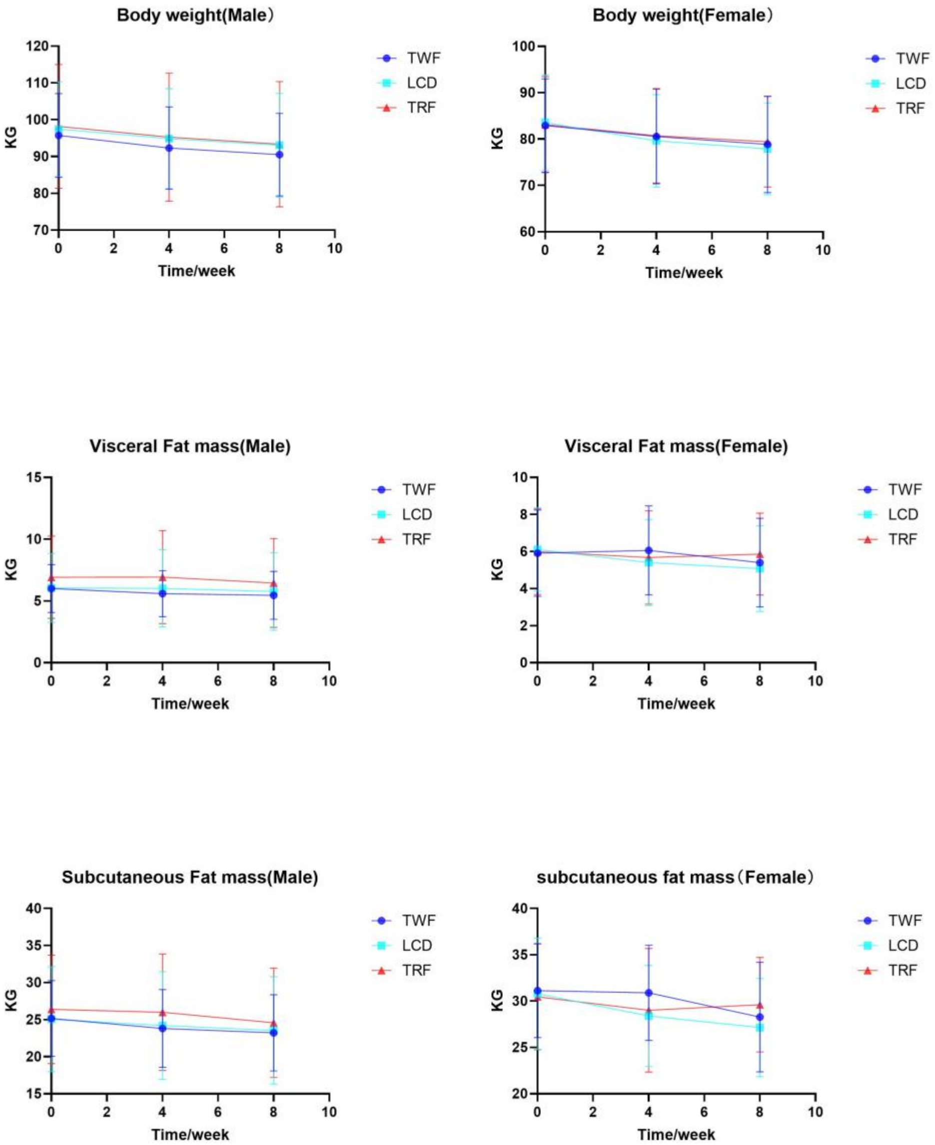

Supplement: Multimedia Appendix 4 [file jmir_v27i1e65868_app4.pdf]
